# “Time is aorta?”: Timeliness of surgical repair in type A aortic dissection

**DOI:** 10.1111/jocs.16412

**Published:** 2022-03-27

**Authors:** Arnaldo Dimagli, Gianni D. Angelini

**Affiliations:** ^1^ Bristol Heart Institute University of Bristol Bristol UK

**Keywords:** aortic dissection, malperfusion

## Abstract

Acute type A aortic dissection is a life‐threatening event that requires prompt management, a complex interaction among the timing of aortic surgical repair, presence or absence of organ malperfusion, and surgical outcomes exists. Whether resection of intimal entry tear should be deferred after reversal of malperfusion and end‐organ ischemia is a matter of controversy. In fact, the timing of aortic repair should be considered within the clinical presentation and baseline characteristics of each patient. Moreover, every effort should be made to minimize times between symptom onset, diagnosis, and surgery.

1

Acute type A acute aortic dissection (ATAAD) is a catastrophic event characterized by an extremely high mortality rate, for which emergency surgical repair is indicated. The rationale for such a prompt strategy relies on preventing lethal complications, such as aortic rupture and tamponade, through the excision of the intimal entry tear in the ascending aorta.

When surgical repair is delayed, the mortality can be as high as 35% in the first 24 h and reach 90% after 30 days,^1^, ^2^ However, even when ATAAD is repaired promptly, the operative mortality can be dishearteningly high, especially in patients presenting with malperfusion and end‐organ ischemia (cerebral, myocardial, mesenteric, and limb), which confers the poorest surgical outcomes.^3^ In international registries collecting data on ATAAD, the overall in‐hospital mortality for surgical patients is around 17% and the incidence of malperfusion syndrome ~35% ^4^, ^5^, ^6^, ^7^

In the current issue of the Journal, Shetty et al.^8^ examine the complex interaction between malperfusion, timing of surgical repair, and operative outcomes of 68 patients undergoing ATAAD surgical repair over a 10‐year period. Malperfusion was present in 42% of patients at presentation and the 30‐day operative mortality was 22%, but significantly increased to 35% in patients with concomitant end‐organ ischemia. In investigating the timing associated with ATAAD repair, authors should be congratulated for prospectively collecting the exact timing of symptom onset, which is usually instead an estimated, derived parameter.

The timing of surgical repair in the context of ATAAD has always been debated and has not yet been fully elucidated. Often, malperfusion and timing are considered independent factors in the clinical decision‐making process, while they should be considered the two sides of the same coin. As shown by Shetty et al., patients seeking medical help earlier were also those more likely to present with malperfusion, whereas the incidence of malperfusion was lower in patients with longer symptom duration and no patients presented with malperfusion after 4 days from symptom onset. Consequently, patients undergoing surgical repair earlier had a significantly higher operative mortality compared to patients with delayed surgery.

In the International Registry of Acute Aortic Dissection (IRAD), malperfusion represented the second most important cause of death after aortic rupture.^1^ Also, it was observed that open aortic repair does not guarantee restoration of distal perfusion as such, and end‐organ malperfusion persisted in up to 25% of the patients, contributing to the dismal operative outcomes.^9^ Based on this, some aortic surgeons have started to advocate delayed repair of the dissection in favor of an earlier reversal of end‐organ ischemia. In a cohort of 602 patients with ATAAD, Yang et al.^10^ investigated the impact of a management plan based on upfront endovascular stenting/fenestration followed by delayed aortic surgical repair for patients with mesenteric malperfusion. This strategy was proven to offer favorable short‐ and long‐term outcomes in this high‐risk cohort.

Hence, how can aortic surgeons provide the best treatment strategy for these patients?

Once again, the focus should be shifted on the clinical presentation and characteristics of each patient rather than just aiming to repair the aorta promptly and quickly. This would imply that some patients may undergo endovascular procedures to restore the organ flow first, and then aortic repair is performed. In this context, risk stratification assumes a central role to guide clinical decisions.

In the last years, three risk scoring systems have been developed to predict 30 mortality risks after surgical repair of ATAAD: the GERAADA,^11^ IRAD,^12^ and UK Aortic^6^ scores. Despite being developed and tested in different cohorts of patients with ATAAD, all identified the unfavorable role of malperfusion in impacting 30‐day mortality. However, further validation and performance analyses are necessary before these scores can play a central role in guiding and outlining the best strategy for each patient. It will be also important to recognize the heterogeneity of “malperfusion,” which entails different target organs (e.g., brain, bowel), characterized by different physiologic degrees of resistance to ischemia. Therefore, more granularity on “malperfusion” and its impact on the outcomes is needed in future reports.

It is important to highlight that in the study by Shetty et al., malperfusion was not found to be a risk factor for 30‐day mortality, and only longer cardiopulmonary bypass times were associated with a higher risk of mortality. This could represent a spurious finding mainly dictated by the small sample size of this cohort and by the limited number of events.

Authors should be also congratulated for shedding some light on the need for standardized referral pathways for ATAAD patients in the reality of developing nations. In western countries, the implementation of efficient referral networks has shown a positive impact on outcomes by reducing the waiting and transfer times.^13^ Moreover, the establishment of specialized aortic services able to provide 24/7 access to these patients and to have a direct contact with the referral centers was associated with further improvement in the morbidity and mortality burden following ATAAD surgical repair.^14^

In the Shetty et al. cohort, only a third of patients were admitted to the hospital within 24 h from symptoms onset, and only 10 patients were offered surgery in the first 24 h. This proportion is strikingly smaller compared to the figures reported in registries from developed countries.^15^ This number is even more worrisome considering that at least a quarter of patients with ATAAD dies before reaching any hospital. In fact, as for most of the surgical registries on ATAAD, also this study suffers from survival bias as only patients surviving and able to reach the hospital were included in the analyses. This could explain why the reported mortality is similar to the ones in other registries despite a higher incidence of malperfusion and longer times between symptom onset and surgery.

One tool to improve these outcomes is to establish programs that increase awareness of this condition so that lag times between presentation and diagnosis, and subsequently referral and surgery, are reduced. Then, building standardized referral pathways within reach tertiary centers with focused expertise would further tackle the fatal outcomes of this condition.

Shetty et al. should be congratulated for presenting these important results and providing food for thoughts on the complex interaction between timing of symptom onset, malperfusion, and outcomes in patients undergoing surgical repair for ATAAD.

## CONFLICTS OF INTEREST

The authors declare no conflicts of interest.
